# Adaptive Management of Protected Wildlife Populations in Poland: Environmental Sustainability and Conservation Challenges of European Bison (*Bison bonasus*), Eurasian Beaver (*Castor fiber*), and Eurasian Moose (*Alces alces*)

**DOI:** 10.3390/ani16131947

**Published:** 2026-06-23

**Authors:** Andrzej Dzikowski, Michał Mierkiewicz, Katarzyna Filip-Hutsch, Blanka Orłowska, Krzysztof Anusz

**Affiliations:** Department of Food Hygiene and Public Health Protection, Institute of Veterinary Medicine, Warsaw University of Life Sciences, 159 Nowoursynowska St., 02-787 Warsaw, Poland; vet.mierkiewicz@gmail.com (M.M.); katarzyna_filip-hutsch@sggw.edu.pl (K.F.-H.); blanka_orlowska@sggw.edu.pl (B.O.); krzysztof_anusz@sggw.edu.pl (K.A.)

**Keywords:** veterinary medicine, law, species protection, nature conservation, public safety, sustainability, European bison, beaver, moose

## Abstract

Populations of European bison (*Bison bonasus*), Eurasian beaver (*Castor fiber*), and Eurasian moose (*Alces alces*) in Poland are currently experiencing significant growth. These species are subject to strict legal protection or specific regulatory frameworks. The purpose of the study is to analyze Polish legislation concerning the protection of selected species and to identify legislative actions that could ensure healthy, sustainable, and well-managed population levels in Poland. The study also explores carefully regulated forms of sustainable use, including the potential consumption of meat from these species. During this research, the methodology of analysis and scientific interpretation of legal acts was used. Case law and relevant socio-economic and environmental factors were also analyzed and highlighted. The results show that the law currently in force and its interpretation may pose challenges to achieving fully effective conservation outcomes. Wildlife protection requires effective, locally adapted population management. Proposals for legal changes that would support diversified and sustainable management approaches, while maintaining a high level of protection, ensuring environmental stability and sustainability, and ensuring the highest standards of public safety, are presented. De lege ferenda postulates indicate that it is essential to balance the legitimate interests of wildlife conservation, public health, and society.

## Simple Summary

The protection of wild animals such as European bison, beavers, and moose requires adaptive management of their populations. Population management should be effective and locally adapted. It is essential to balance the legitimate interests of wildlife protection and conservation, public health, and the economic and non-material interests of society. One of the tools for achieving this goal is the law. In the context of the current status of European bison, beaver, and moose populations in Poland, this paper analyzes their legal protection and proposes potential legislative adjustments to support sustainable population management. The study also considers, under specific and strictly regulated conditions, the potential use of meat from these species while maintaining biodiversity conservation, environmental sustainability, and high standards of wildlife management.

## 1. Introduction

Populations of European bison (*Bison bonasus*), Eurasian beaver (*Castor fiber*), and Eurasian moose (*Alces alces*) in Poland are currently experiencing a significant growth in terms of the total numbers of individuals, and the expansion of their inhabited areas. This trend is closely linked to long-term conservation efforts and successful species recovery programs [1,2,3,4,5,6,7,8,9,10].

The European bison, once extinct in the wild in the early 20th century, has been restored through coordinated international breeding programs based on a very limited number of founding individuals [11,12,13]. Poland has played a central role in this process, particularly through breeding centers and free-ranging herds established in areas such as the Białowieża Forest and Bieszczady Mountains [2,11]. Over time, reintroduction efforts have expanded the species’ range beyond its historical refuges, contributing to the gradual stabilization and growth of the population [1,11]. Currently, Poland hosts the largest free-ranging population of European bison in the world, numbering about 3000 individuals [1,2], distributed across several regions, including the north-eastern, eastern, and, increasingly, western parts of the country.

The Eurasian beaver, which had nearly disappeared from much of Europe due to overexploitation, began to recover in Poland in the mid-20th century through strict legal protection and active reintroduction programs. Since then, the species has demonstrated a strong capacity for natural expansion, recolonizing river systems and wetlands across the country [4,7]. Today, the beaver population in Poland is estimated at over 100,000 individuals and is widely distributed across most river basins, making it one of the most widespread large mammals in the country. Its population growth is often cited as one of the most successful examples of wildlife restoration in Europe [4,5,6,7].

The Eurasian moose population in Poland has also undergone significant changes over time. After periods of decline caused by habitat loss and human pressure, the introduction of protective measures, including a long-term moratorium on hunting, allowed the population to recover [9,10]. Favorable environmental conditions and the species’ ecological adaptability have further supported its expansion into new areas [9], including regions with increased human presence. The current population is estimated at about 30 thousand individuals, with the highest densities observed in north-eastern Poland [3,9], although the species is increasingly present in central and eastern regions as well.

Due to the limited availability of natural environmental resources, such as forests and grasslands, interactions between wildlife and human activities and land use are inevitable and often complex. Moose may affect transport safety and forest regeneration; beavers modify water systems, influencing agricultural or recreational areas and infrastructure; and European bison may cause damage to crops and forest plantations and, in rare cases, pose challenges in human–wildlife interactions [3,4,5,9,14,15,16,17].

Resolving such conflicts and managing wildlife populations is complex. Polish law establishes strict limitations on activities involving protected animal species, and violations may result in legal consequences [18]. Current legal provisions may also limit the flexibility of adaptive management of these populations. In practice, this means that killing or disturbing protected species is generally prohibited, with only limited exceptions permitted under Polish law, including cases related to public safety, scientific purposes, prevention of serious damage, disease control, or justified population management measures [18,19]. Populations of several protected mammal species in Poland have increased substantially in recent decades. Current estimates indicate that the European beaver population exceeds 150,000 individuals, whereas the moose population is estimated at approximately 28,000–30,000 individuals, although these figures should be interpreted cautiously because both species are difficult to survey accurately [4,7,9,17]. In some regions, increasing population densities are associated with growing ecological impacts and socio-economic conflicts, including agricultural and forestry damage. Therefore, carrying capacity should be considered not only in terms of habitat productivity but also in relation to social acceptance and acceptable levels of economic losses [4,9,10]. Since the populations of the discussed species are beginning to exceed local ecological and socio-economic limits, it is worth considering whether their protection can be maintained alongside selected forms of sustainable use, including the carefully regulated consumption of meat from these species. Similar experiences from Europe show that the recovery of formerly depleted wildlife populations may necessitate adaptive management strategies. The restoration and expansion of beaver populations across Europe, together with proposed approaches for sustainable management of the increasing moose population in Poland, suggest that species conservation and carefully regulated utilization are not necessarily mutually exclusive [9,10,15,16]. 

The aim of the current paper is to analyze, on a comparative legal basis, Polish legal norms regarding the conservation and management of European bison, moose, and beaver populations. The study aims to provide a legal analysis focused on identifying threats and highlighting positive aspects, and to identify legislative actions that could ensure healthy, sustainable, and well-managed population levels of the species in Poland while maintaining a high level of nature conservation. One possible approach is to consider regulated forms of resource use while simultaneously ensuring the highest standards of public and environmental safety. The fundamental assumption underlying this research is that wildlife conservation law can be adapted to balance human activities with the sustainable protection of wildlife. Such an adaptive approach is essential for achieving effective, long-term conservation outcomes.

## 2. Materials and Methods

Polish [18,19,20,21,22,23] and European [24,25,26,27,28,29,30,31] legal acts concerning species protection, the admissibility of certain forms of wildlife use, and public safety standards were examined. The obtained results were compared with those from the analysis of the legal frameworks of European Union member states and selected third countries [32,33,34,35,36,37,38,39,40] and with relevant provisions of international law [41,42]. Scientific methodology for the analysis and interpretation of legal acts was used, including linguistic, systematic, functional, teleological, European, and comparative methods of legal analysis and interpretation. Analysis of case law from Polish and European courts and administrative authorities regarding the protection of the discussed species was also conducted [19,43,44,45]. Postulates de lege lata and de lege ferenda were presented. The obtained results were compared with the extralegal conditions of the analyzed problem.

The choice of species discussed in this paper is not incidental. The authors selected them for several reasons. The European bison is a well-established example of successful nature conservation and an iconic species within Poland’s fauna. Moreover, it is subject to strict protection and dedicated active conservation programs.

The Eurasian beaver is also protected under Polish law, though within a less restrictive regulatory framework than for the European bison, with greater emphasis on passive protection measures.

The Eurasian moose is classified as a game species; however, due to a long-standing prohibition of hunting, its population is currently managed under a regime functionally similar to strict protection.

## 3. Results

The conducted research made it possible to identify the legal basis, as well as axiological and teleological foundations, underlying the level, methods, and consequences of the protection of European bison, beaver, and moose in Poland. The analysis indicates that the current legal framework for the protection of wild animal species in Poland is well-developed at the formal level (“law in books”), although certain challenges may arise in its practical implementation (“law in action”). Consequently, the effectiveness of nature conservation and population management may vary in practice. At the same time, the causes of this state of affairs were identified, which lie in the very idea of classical species protection, and are closely related to the particular importance of the species in question for Polish nature and society. The paper also outlines potential directions for developing more adaptive approaches to wildlife population management, including regulated forms of resource use and carefully supervised meat consumption, while maintaining a high level of species protection and public and environmental safety. A comparative overview of selected ecological characteristics, conservation benefits, management challenges, and socio-economic impacts associated with European bison, Eurasian beaver, and Eurasian moose populations in Poland is presented in Table 1.

### 3.1. Current Status of Species Legal Protection

The basic legal act regulating the protection of wild animal species in Poland is the Nature Conservation Act [18]. This statute is accompanied by implementing provisions [22] and by European and international law [24,25,41,42].

Among these, the Habitats Directive is particularly important, as it significantly shapes many aspects of species protection across Europe [24]. The Directive establishes different protection regimes depending on species classification. In particular, Annex IV provides for strict protection, including a prohibition on killing, while Annex V allows for the controlled use of certain species. It was also observed that the Directive is formulated in relatively general terms, thereby allowing for a degree of legislative and decision-making flexibility at the level of individual Member States.

#### 3.1.1. European Bison

Habitats Directive Annexes II and IV [24] list the European bison as a priority species, imposing special obligations on Member States to protect it, including protection of its habitats. Thus, the Natura 2000 network areas are designated as mandatory. Due to potential threats (such as low genetic variability, diseases, and population isolation), active conservation measures are implemented [18,24,41,42].

It was revealed that in Poland, the European bison is strictly protected under Art. 49 of the Nature Conservation Act and §§ 1–2 of the implementing regulation [18,22]. These regulations implement Art. 12 of the Habitats Directive [24].

Intentional killing, capture, keeping, trade, and sale of bison are strictly prohibited, and penalized with a fine, restriction of liberty, or imprisonment [18,22,24,41,42].

Possession of any products from the European bison is illegal without a special derogation permit and is prohibited by law [18].

Strict species protection for the European bison does not mean absolute, unconditional protection under the law. Exceptions and derogations are possible under Art. 56 of the Nature Conservation Act [18]. Exceptions under this rule are equivalent to exceptions under Art. 16 of the Habitats Directive [19,43,44,45].

In justified cases, such as disease, injury, damage, or a threat to human safety, it is allowed to eliminate individuals. Sanitary or reduction culling as part of population management is acceptable, as a derogation from species protection prohibitions. These actions must be strictly monitored, controlled, and reported. They may only take place after obtaining the individual administrative consent of the General Director for Environmental Protection, in accordance with Art. 52 and 56 of the Nature Conservation Act [18,52]. Blanket consents are not issued. Only the elimination of individual European bisons is permitted for specific reasons permitted by the law, and proven in administrative proceedings.

Analysis of the case law of administrative authorities and courts [19,45] regarding the admissibility of derogations from the normative prohibition on killing European bison allowed for the following results to be obtained. The presented results can be applied, mutatis mutandis, also to the remaining animal species studied, especially to beavers.

The justifications accepted and recognized as lawful by the General Director for Environmental Protection and administrative courts allow the application of the procedure under Art. 56 of the Nature Conservation Act [18,52] included: sicknesses, injuries, or aggressiveness of the animal; an important public interest; damages to agriculture; an increase in the European bison population outside forest areas; lack of alternative solutions with no negative impact on the species’ population.

Administrative courts have developed several key rules. Primarily, the culling of European bison must be treated as a strict exception, yet it is legally allowed in practice. The analyzed case law primarily confirms the classic model of applying Art. 56 of the Nature Conservation Act [18], i.e., culling as a tool for population protection, not population reduction. It was noted that the law always prioritizes population protection over individual European bison protection. Courts have clearly indicated that eliminating individual European bison can serve the species as a whole. Judicature arguments emphasize preventing animal disease and suffering [19].

Moreover, as indicated, damage to agriculture and forestry was also considered an acceptable ground for derogation. Administrative bodies and courts accept the application of Art. 56 of the Nature Conservation Act based on such factual grounds. This implies allowing the reduction culling of European bison for economic reasons, not only health reasons. At the same time, the Supreme Administrative Court [45] emphasized the need for a reliable analysis of the impact on the local European bison population.

According to the judicature, the administrative authority (General Director for Environmental Protection) must justify the decision, demonstrating specific threats as its grounds, a lack of alternative solutions, and simultaneously determining the impact of the culling on the European bison population [18].

It was revealed that in practice, “limit” administrative decisions, e.g., “up to 20 European bisons”, are permissible. However, they are controversial and have been challenged in court [45]. In such cases, the rule is that the administrative court requires a solid scientific justification of the administrative derogation decision. The analyzed regulations do not require individualization of each animal. In the judicial opinion, it is important to determine the maximum number of European bison and the conditions for implementing the decision, rather than to identify specific individuals. Each decision must be based on reliable evidence, including scientific data on the European bison population, and must include an analysis of the impact of culling on the species’ status [45].

As a strictly protected animal, the European bison is not a game animal [19]. The legal consequences are the lack of designated hunting seasons and the inability to hunt bison legally or obtain bison meat. It was found that European nature protection law, including the Habitats Directive, does not prohibit the use of meat from legally culled protected animals [24,43].

#### 3.1.2. Beaver

Under European and national law, beavers are a protected species, but this protection is not as strict as in the case of European bison [18,22,24]. This species is included in Annexes II, IV (excluding the Polish beaver population), and V (including the Polish beaver population) to the Habitats Directive [24]. This model is referred to in Polish law as “partial protection”. Its basis is the prohibition on killing, disturbing, and destroying the habitats of protected species [7,15,18]. Nevertheless, it allows culling, trapping, or destruction of beaver dams—while maintaining the administrative procedure for obtaining a derogation permit. Such a permit, issued by the Regional Director for Environmental Protection, can be granted for a specific area for up to 5 years and is based on Art. 52 and 56a of the Nature Conservation Act [18]. It was revealed that in practice, such decisions are very common in Poland, issued relatively easily and on a mass scale (usually as “limit” decisions). The actual justification for these decisions is most often damage to road and water infrastructure (flood embankments) and agriculture, thus, economic reasons.

#### 3.1.3. Moose

Moose is not a protected animal species under the Polish Nature Conservation Act [18,22] nor the Habitats Directive [24]. On the contrary, it is a game animal in accordance with the provisions of Art. 5 of the Hunting Law, 1995 [19]. However, in 2001, a so-called “moratorium” was introduced by a regulation of the Minister of Agriculture [30], setting the permissible hunting period for moose in Poland at 0 days per year. Thus, through a sub-legislative act, de facto full, passive protection of this species was introduced. Therefore, the Polish model of moose population management can be described as a protective model or as an example of a precautionary approach focusing on animal protection. The lack of a hunting season excludes moose from the hunting economy and management. Due to the lack of legal possibility of hunting, there is no legal supply of moose meat in Poland (except for imports).

## 4. Discussion

### 4.1. Factors Affecting Status and Scope of Legal Protection: Legal Protection’s Impact

The legal protection status of a species significantly affects its abundance and population stability. Changes in the legal framework without a well-thought-out strategy and inadequate legal responses lead to errors in conservation or population management strategies. The fundamental act is the Habitats Directive [24], which affects the level of protection afforded to various species across countries in diverse ways. It was revealed that, under Polish law, these manifestations include: a precise reflection of the Directive’s protection regime for European bison, and a slight tightening of the protective regime for beavers. Moreover, a somewhat more restrictive approach to the practical management of moose was identified.

The Directive [24] is not a normatively general act, but a functional framework. It precisely defines the levels of species protection but leaves particular countries broad discretion in applying exceptions. The current analysis shows that the directive is not overly general. Its structural precision is accurate, as it clearly distinguishes between levels of protection (Annexes IV vs. V), specifies obligations (Art. 12), and defines the grounds for exemptions and derogations (Art. 16). However, it does not specify how many animals can be culled or when exactly the damage is “sufficient”. It leaves a wide margin for national legislation, causing international differences in species protection and legal disputes. It is this legislative and decision-making discretion that leads to disputes over culling European bison or regulating beaver populations.

Analysis and interpretation of legal norms led to the conclusion that the main axiological basis for all these regulations, and for their predominant interpretation, is the classic conservation approach, which primarily requires the preservation of all individuals of a given endangered animal species [53]. This is directly related to the status of endangered species populations, which were drastically low at one point in history, and later, through various legal and extralegal measures, were significantly rebuilt and increased. This observation applies to all three species studied.

However, it was discovered that the applicable law [18,24] also allows the adoption of other, more advanced and unconventional strategies for protecting species. This relates to the more advanced methods of interpreting legal provisions, which hold that the goal of conservation is to ensure healthy, well-adapted, and self-sufficient populations. Species protection laws inherently require protection through adaptive management and prioritize the quality of wildlife populations over the quantity of individuals. Only some interpretations adopted by administrative authorities or judicial bodies are based on an older paradigm and a stricter, fragmentary approach.

### 4.2. Advantages and Disadvantages of the Current Model

The research shows that the basic factors influencing the protection status and the potential forms of wildlife use animals are the population size, health status, conservation status, ecological characteristics, and stability of the local population. This requires a legal model of protection, which should be derived from and consistent with the scientific model. However, the findings of this study indicate that such alignment may not always be fully reflected in the current Polish framework for the three species discussed. A comparative analysis with other countries and models of wildlife protection and management further suggests that there may be differences in the extent to which this approach is implemented in Polish law. For example, Polish law does not currently provide regulations addressing the ongoing, often sensitive discussion of approaches that could permit controlled meat consumption within a clear legal framework for these three species. The history of the extinction of the European bison in the wild and its subsequent reintroduction is well known [11,12,13,14,54], but beavers and moose also almost completely went extinct in Poland in the second half of the 20th century [3]. As a result, conservation strategies were aimed directly at restoring these populations and protecting ecological and cultural heritage [8,12,13]. In the discussed triad of species, the success of the protection system introduced by the law and the recovery of the population from its almost complete extinction to a numerous, dynamically developing population are evident [8,9,55,56]; the European bison evidently highlights this. Currently, changes in the number of wild animals of the analyzed species have not influenced the shape of the legal norms, which remain based on the old scientific paradigm.

The following paragraphs present the positive aspects of species conservation, along with selected challenges associated with the current protection model.

#### 4.2.1. European Bison

The European bison population in Poland is estimated at ca. 3000 animals, and its status is defined as near threatened [1,2]. Free-living European bisons are conservation dependent [1,2]. European bison live in Poland in the wild and in special breeding enclosures; currently, there is no commercial breeding of either European bison or the other species analyzed.

The recovery, restoration, and reintroduction of the European bison population have been a great success, perhaps the greatest in the history of nature conservation [11,12,13,14]. This success is currently being discussed, and the European bison population in Poland seems to be the victim of its own success.

The problems revolve around several issues: excessively large herds and excessive density to the ecological carrying capacity of a given habitat, and compromised environmental sustainability. Simultaneously, population fragmentation is significant. Furthermore, small herd sizes pose a risk of being easily wiped out by transmissible diseases [57]. The low genetic variability of this species remains an insurmountable problem. This means that bison conservation is not a complete process but requires ongoing monitoring and human involvement [1,11].

In this context, any population regulation measures require particular caution. Selective removal of individuals may influence population structure, social organization, and the already limited genetic diversity of the species, especially if mature and reproductively important individuals are disproportionately affected [11,13]. Therefore, management decisions should be based on long-term genetic and demographic monitoring [1,45].

The current conservation model for European bison is not entirely satisfactory. Specific recommendations for the future include creating metapopulations, translocations, gene banks, and adaptive management [1,13]. Deepening the integration of in situ and ex situ management options, as well as standardizing strategies across countries with European bison populations, is necessary.

European bisons adapt to new conditions, which is important for assessing the success of conservation measures and has significant implications for population management [14]. Simultaneously, such plasticity and area expansion imply a greater frequency of conflicts with humans.

An important consideration in discussions on potential changes to the current conservation model is the symbolic role of the European bison as a restored species of national significance, alongside the influence of established protection approaches. At the same time, a balanced integration of cultural and scientific perspectives appears essential.

#### 4.2.2. Beaver

The size of the beaver population in Poland is difficult to estimate; there is no confirmed, complete, and current data. There is no doubt, however, that this population shows very strong and significant growth and an increase in its territorial range. After World War II, there were about 100 beavers in Poland; in the 1970s, ca. 1000; and in 2021, the population was estimated at ca. 148,000 individuals [3,4,5,6,7,8].

The strong increase in the beaver population is the result of planned reintroduction activities (introduction, settlement, resettlement) and the enforcement of partial legal protection, including prohibitions on hunting and on the destruction of dams and lodges [4,6,7,15,18,48]. Thus, beavers are relatively easy to encounter, as they inhabit a wide range of watercourses across Poland, including those located in urban areas. This may be seen as evidence of the species’ high ecological adaptability.

The relatively high density of beavers, together with their capacity to shape habitats and ecosystems [5,48], can pose challenges, including tree felling, localized flooding, and impacts on embankments and infrastructure [4,6,8,15,16]. At the same time, the undeniable benefits of beaver activity are evident, such as water retention and the creation of new habitats for other species (resulting in increased biodiversity and environmental heterogeneity). At the same time, population management measures affecting beavers may also influence local hydrological processes, habitat connectivity, and ecosystem functions shaped by beaver activity. Therefore, management decisions should take into account not only damage prevention but also the ecological role of the species as an ecosystem engineer [5,16,47]. It should be noted that these ecological, biodiversity, and climatic factors are among the pivotal utilitarian motivations for beaver protection [5,8,47,49]. Currently, the state authorities are taking action to counteract the significant damage caused by beavers, primarily by eliminating beavers (de facto hunting), destroying lodges (derogations through an act of local law), and securing drainage ditches, flood embankments, and trees [3].

#### 4.2.3. Moose

Similarly, moose is an example of high species plasticity and a very rapid, large population recovery. According to available official estimates, the population in Poland currently exceeds 30,000 individuals, representing a substantial increase compared to the early 21st century, when it was estimated at below 2000 individuals [3,9,10]. This growth is an obvious effect and an indisputable result of the introduction of the “moratorium” ban on hunting [9,17,50].

The recovery of the moose population has brought several positive outcomes, including the restoration of a key native species, the strengthening of ecosystem functions (such as the natural shaping of vegetation structure), and an increase in biodiversity in wetland and forest habitats. In addition, the improved population structure, including a higher proportion of mature individuals such as well-developed bulls with characteristic antlers, may be observed, which can be interpreted as an indicator of favorable habitat conditions and reduced anthropogenic pressure.

At the same time, any future population regulation measures would require particular caution. Selective culling may affect population structure, sex and age composition, and the presence of mature individuals, which could influence long-term population dynamics and ecological stability [9,17,50].

Moose are occupying territories across the country that are growing wider, and they are increasingly often coming into conflict with humans. As a result of a significant increase in the moose population, new problems have emerged, primarily the risk of road accidents, which often result in fatalities for both humans and animals [9,17]. Furthermore, higher moose population densities may affect agriculture and forestry [10,17,51].

Current governmental measures include the use of mechanical, agrotechnical, and chemical methods of indirect crop protection, as well as actions aimed at improving habitat conditions for moose and managing their habitats (e.g., establishing browsing plots or designating wildlife refuges) [3]. While these approaches primarily focus on mitigating impacts on agricultural vegetation, their effectiveness may vary depending on the scale and consistency of implementation, as well as the extent to which they address broader aspects, including human safety.

#### 4.2.4. Social Influence and Attitude

All three species discussed may have impacts that shape societal perceptions of their protection. In each case, it is possible to identify a general trajectory in human–wildlife relations: from past population decline, through successful conservation and recovery, to present-day challenges associated with growing populations and wider distribution [6]. Growing animal populations can affect agriculture and forestry and may increase the risk of certain incidents, with potential social and economic consequences [9]. This, in turn, can shape public perceptions and approaches to protected species [6]. The discussed animals are also a major tourist asset, being a key aspect of local tourism. At the same time, public attitudes toward these animals may be ambivalent, as they are perceived both as valuable elements of natural heritage and as sources of certain challenges [5,6,8]. Thus, social tension between species protection and the need to control it is emphasized. In the authors’ opinion, this is a crucial extralegal factor in species protection that simultaneously influences future legal norms.

Managing conflicts and damages is a key element of effective nature conservation strategies. Preventing future conflicts requires flexible, locally tailored solutions rather than standard conservation methods [58].

At the same time, further empirical research on the socio-economic impacts of these species, including public attitudes, stakeholder perspectives, and the economic consequences of wildlife-related damage, would be valuable for the development of future adaptive management strategies. A comparative overview of selected ecological characteristics, conservation benefits, and management-related impacts is presented in Table 1.

#### 4.2.5. Comparative Legal Aspect

In light of the Directive [24], it is not possible to exclude the European bison entirely from the system of species protection or to classify it as a game species [53]. However, this does not preclude the possibility of limited and strictly regulated interventions under specific conditions.

In the case of beavers, the situation across individual European countries ranges from recognizing it as a game animal to controlled population regulation to providing at least partial legal protection [16]. Conservation status and hunting eligibility of beavers are directly related to local population size.

A comparative analysis shows that the lack of detailed regulation of moose in European Union law results in significant variation across national models. States have a wide margin of discretion in this regard, allowing them to adapt hunting policies to local ecological and economic conditions.

As noted, the so-called “moratorium” on moose hunting was introduced in Poland in 2001 by a regulation of the Minister of Climate and Environment [22]. Under the Hunting Law [19], the species remains formally classified as a game animal. The relevant statutory provisions (Art. 4 and 5) indicate that hunting policy should be based on the management of animal populations. In this context, questions may arise as to the scope of the Minister’s authority to determine hunting seasons, including the possibility of setting them at a level that effectively precludes hunting.

From a legal perspective, the moratorium may be subject to differing interpretations, particularly regarding the extent of the delegated powers under the statute. As a result, the current approach to moose protection operates within a framework that differs from a model based on explicit statutory designation of strict protection.

Furthermore, moose are not explicitly protected under the Nature Protection Act. This means that the current system of their protection is based mainly on secondary legislation rather than a direct decision of the legislature. As a result, the approach adopted at the ministerial level may differ from the solutions that would follow from clear statutory regulation. On the other hand, hunting law is traditionally regarded as one of the instruments of nature conservation [59]. Moreover, in light of the Directive [24], Member States are required to ensure a favorable conservation status of species, which may justify the introduction of a hunting moratorium, particularly in view of the condition of the moose population in the early 2000s. At present, however, the continued application of this measure may raise questions as to its proportionality and legal basis in light of current population trends. From a legal perspective, the long-term maintenance of a de facto total suspension of hunting invites further discussion regarding its consistency with the statutory framework.

This is also an exceptional situation compared to other European countries, especially the neighboring ones [9]. In the Scandinavian and Baltic states, in Russia, and in Belarus, the moose population is large; therefore, moose are intensively hunted, including for meat [60].

Sweden manages moose populations through hunting [32,33]. It is a decentralized, adaptive management system. Game shooting is the primary tool for population regulation. This system assumes that hunting is an integral part of natural resource management. Therefore, moose hunting is permitted, but requires meeting specific organizational and planning conditions. In particular, hunting takes place during designated seasons, in designated locations, and in accordance with management plans, which are subject to administrative approval. According to Art. 33 of the Swedish Hunting Law [32], moose hunting may take place based on a permit issued by the County Administrative Council and in an area registered by this board (so-called licensed area), also without a permit in a registered area (so-called moose management area). The area must be suitable for moose hunting and of sufficient size to allow the shooting of at least the number of adult moose specified by law per year [32]. There must be a management plan for the discussed areas [32,33].

At the same time, Swedish regulations also provide for an institutional model of co-management—moose management groups consist of representatives of landowners and hunters, and their task is to develop culling plans that align with conservation and rational management goals [32]. The inclusion of public participation in the legal regulations is highly praiseworthy and should serve as a model for other hunting legislation.

In Norway, §§ 16–18 of the Hunting Law [37,38] provide for local resource management that takes into account the possibility of economic exploitation of wild fauna, including hunting. The central Administrative Directorate determines which hunting areas may be conducted, as well as the minimum areas or quotas for the felling of animals. Local municipal authorities issue specific felling permits. All decisions should be based on the size of the moose population, its living conditions in the particular district, and the damage caused by moose [37]. Hunting can be differentiated by individual animal characteristics (e.g., gender or age) [37], allowing for more precise population management. Therefore, in Norway, moose hunting is closely linked to local population conditions and is flexible. This model is an example of advanced decentralization, with local authorities playing a key role.

The Finnish model involves administrative and scientific regulation and central administrative control of moose population management (a controlled system in which hunting is strictly controlled and planned at the national level) [35,36]. The Finnish Wildlife Agency issues cervid hunting licenses. This administrative authority, in granting licenses, shall ensure two crucial goals: that the local moose population is not endangered by hunting, and that hunting will keep damage caused by moose to traffic, agriculture, and forests at a reasonable level. Furthermore, opinions of regional stakeholders and the strive for a fair distribution of hunting opportunities must be taken into consideration. It should be noted that § 26 of the Finnish Hunting Law [36] explicitly links the permissibility of hunting with the need to maintain a balance between species conservation and economic interests, which is the essence of the discussed model. Moose population management models in these countries vary and lack uniformity, but all respond to the current state of the moose populations in their areas. Scandinavian countries actively use legal instruments to shape the population size of this species. They implement a model of active population management that relies on hunting as its primary regulatory tool. The Baltic states, on the other hand, also regulate moose hunting and management, but have lacked long-term plans. Remedial measures are implemented only where problems such as forest damage arise. Compared to countries like Sweden and Finland, which have systematic hunting laws and management strategies, the Baltic states have not developed a stable legal framework to ensure sustainable management of the moose population [60].

Poland faces a similar challenge, shaped by the limited use of specific tools for moose population management. Current approaches rely mainly on non-lethal measures, which support species protection, but their ecological effects should be regularly monitored. At the same time, any more intervention-based actions would require particular caution, as they could affect population stability and genetic diversity, especially by impacting mature individuals.

Therefore, conservation policy should clearly prioritize the long-term protection of the species, maintaining a favorable conservation status and stable population structure. Future decisions should be based on scientific evidence and a precautionary approach, ensuring that the sustainability of the moose population is not compromised [9,50].

All the presented approaches are consistent with European law, but they lead to different practical consequences—from practical full species protection to its controlled use as a natural resource.

When it comes to the European bison, the primary animal with which it can be compared is its closest relative—the American bison (*Bison bison*). The legal status of the American bison in its native range is pluralistic. It might be considered a protected species (“non-amenable”/“exotic” status) and might be subject to hunting in the wild. Still, it is also kept on farms, which encompass the majority of the species’ population. The situation of American bison in North America depends on federal, state, location, and ownership context. The legal status and recognized methods of population management, therefore, differ significantly between European and American bisons. Hunting and slaughter of farm-raised bison for meat are permitted in both the United States and Canada [39,40]; this meat is consumed domestically and exported to Europe.

American bison are also kept in Poland and other European countries, and slaughtered for human consumption [26]; the same applies to the European bison farmed in Sweden or Germany [61,62]. It should be noted that, from the point of view of veterinary epidemic law, European bison kept by men are treated in the same way as farmed American bison or cattle (all being “bovine animals”) [28,31,57]. In addition, breeding American bison in Poland requires strict supervision, not only veterinary, but also protective, due to the risk of epidemics, to ensure these animals do not endanger or contaminate the native population of European bison.

Another species that can be compared to the European bison is the chamois (*Rupicapra rupicapra tatrica*). It should be noted that the legal status of this species in Poland is comparable to that of the European bison—both are strictly protected under the same legal standards [18,22].

This example, however, demonstrates that the global status of the species does not reflect the situation of local populations. Chamois’ population status can be compared to that of the European bison. Yet, its situation is the opposite—in Poland, there is a relatively large local European bison population (though small on a global scale), and an extremely small and endangered Tatra chamois population (compared to relatively large and stable Alpine and Balkan populations) [63,64]. Moreover, it is necessary to note the habitat fragmentation, isolation, and genetic distinctiveness of chamois in the Polish and Slovak Tatra Mountains. These factors make the population particularly sensitive to environmental pressures and human disturbance, and highlight the importance of maintaining habitat connectivity and minimizing external impacts. In this context, conservation measures should place special emphasis on preserving genetic diversity and ensuring the long-term viability of this unique, transboundary population [63,64,65]. Given this population status, strict protection of chamois in the Tatra Mountains is necessary, and any plans to hunt them cannot be permitted or justified. In different regions of the Alps and the Balkans, chamois are hunted [66,67,68,69], and there are also chamois breeding farms [70].

In the case of chamois, the scientific approach and results of population research find their counterpart in applicable law [18,22]. The essence of species protection in this case is to prevent the extinction of a rare species population, and the uninterrupted legal protection of chamois in Poland dates back to the 19th century [71].

This indicates that the analyzed regulations do not necessarily result from the adoption of a particularly restrictive absolute protection model in Polish law. Rather, it suggests that in some cases species protection measures align with a species’ population status, whereas in others this alignment may be less evident, including for the three species analyzed in this study. In the authors’ view, it appears desirable for legal frameworks to more consistently take into account the current population status of the species concerned.

### 4.3. Conditions of Wildlife Protection and Sustainable Use of Animal-Derived Resources

#### 4.3.1. Non-Legal Conditions of Wildlife Protection and Use of Animal-Derived Resources

The ethical aspects of wild meat consumption are not the primary focus of this study. However, meat obtained through legally justified wildlife management is increasingly discussed in the context of sustainability and “zero-waste” approaches. This is particularly relevant in cases where animals—such as European bison—must be eliminated for sanitary reasons, including disease control. In some instances (e.g., localized parasitic infections such as thelaziosis), the meat may remain suitable for human consumption following proper veterinary inspection. Nevertheless, under Poland’s current legal framework, such meat is typically disposed of, regardless of its potential for reuse. This raises concerns from the perspective of sustainable and rational resource management, although it remains subject to broader ethical and societal debate. Another important non-legal factor concerns human–wildlife interactions. As wildlife populations increase and expand their ranges, interactions between protected species and human activities may become more frequent. This can contribute to more diverse public attitudes toward species protection, including both support for conservation goals and concerns related to economic or safety considerations. In some contexts, this may also lead to discussions on possible adjustments to existing protection frameworks.

From a scientific perspective, research on wild animal products, including meat, has examined aspects such as nutritional composition and quality. Studies indicate that meat from species such as the European bison or beaver may exhibit favorable nutritional characteristics, including relatively low fat content and a beneficial fatty acid profile. At the same time, the relevance and applicability of such findings depend on broader regulatory, environmental, and societal contexts [72,73,74].

The role of wild meat also differs significantly across regions. In some parts of the world, it constitutes an important component of subsistence, while in more affluent contexts, it is more often associated with niche or traditional uses. In the European context, including Poland, any potential use of such resources would likely remain limited and subject to strict regulatory conditions, reflecting both conservation priorities and food safety standards. References to historical culinary practices may illustrate cultural continuity; however, their contemporary relevance should be considered with caution [74,75,76,77,78,79].

Any consideration of the use of wildlife-derived products must therefore take into account a wide range of factors, including conservation objectives, legal constraints, enforcement capacity, and potential risks such as illegal sourcing or overexploitation. In this context, the role of public authorities remains crucial in ensuring compliance with applicable regulations and in safeguarding both species protection and broader environmental interests [18,19,20,21,22,23,27,29,30].

#### 4.3.2. Legal Conditions of Species Protection

The history of Polish nature conservation law reflects early forms of organized species and habitat protection [10,11]. These regulations, however, were primarily motivated by economic and political considerations.

Contemporary law continues certain traditions but is based on broader ecological and social foundations. Art. 56 of the Nature Conservation Act [18] provides a legal basis for exemptions from strict protection, allowing a degree of flexibility in practice, subject to judicial review [19,45]. Such derogations may apply, for example, to cases involving public safety, scientific research, disease control, prevention of serious damage, or justified population management measures, subject to statutory and administrative requirements. The case law of the Court of Justice of the European Union further refines this framework. Derogations under the Habitats Directive must be interpreted strictly, require clearly demonstrated and specific circumstances, and be supported by scientific data, while not adversely affecting the favorable conservation status of the species [24,43,44]. In this context, the application of derogations at the national level may raise questions as to their consistency with EU standards.

At the same time, differences in judicial roles should be noted. Polish administrative courts primarily assess legality and procedure, whereas the CJEU focuses on the effective implementation of EU law objectives. The above considerations concern the legal framework of species protection and possible interventions. A separate, though related issue is the legal and sanitary handling of animal products resulting from such actions.

### 4.4. Food Safety and Legal Conditions of Use

Under current Polish law, meat from European bison, beaver, and moose is generally not traded due to species protection regimes and hunting restrictions [18,19]. At the same time, meat from comparable species, such as American bison or imported game, may be legally marketed.

The introduction of meat into the market requires lawful acquisition and compliance with veterinary and sanitary standards [25,26,27]. Veterinary inspection procedures for game are well established, although species-specific risks must be considered. In particular, the risk of *Trichinella spp.* infection in beaver meat necessitates mandatory testing [31,80]. Other zoonotic risks may also be relevant in the context of wild animal meat.

Available research does not indicate significant scientific contraindications to the use of meat from legally obtained animals, provided that all applicable safety and veterinary standards are met. However, such considerations remain secondary to conservation objectives and legal constraints.

Environmental sustainability requires a comprehensive, ecosystem-based approach. In certain contexts, it may also include considerations related to the management of wildlife-derived resources, provided that conservation objectives are not compromised. At the same time, practical aspects, including potential sanitary risks associated with animal carcasses, should also be taken into account.

### 4.5. Future Possibilities of Adaptive Management of Protected Wildlife Populations

Scientific literature emphasizes that contemporary nature conservation pursues multiple objectives, including environmental sustainability, public and animal health, and the reduction in human–wildlife conflicts [10]. Addressing these challenges requires flexible, locally adapted approaches that account for population size, habitat conditions, and genetic factors [58,64,81].

Modern conservation increasingly focuses on the quality and condition of populations rather than solely on their size, emphasizing health, genetic diversity, and long-term viability [10,81,82,83]. Adaptive management reflects this approach, combining legal instruments with scientific knowledge. Available methods include population monitoring, habitat management, restoration measures, and actions to maintain genetic diversity [2,9,17,58,83].

In some cases, population regulation measures may also be considered within the framework of applicable law, provided that they are based on scientific data and do not compromise the conservation status of the species [10,13,59]. At the same time, management decisions should take into account broader ecological processes, including climate change and natural population regulation by predators [9]. The shift from single-species protection toward ecosystem-based approaches is increasingly emphasized in the literature [58,83,84].

At the same time, any consideration of expanding the use of products derived from protected species requires particular caution. Potential risks include the misuse of derogation mechanisms, difficulties in effective monitoring and enforcement, and possible negative effects on public perception and social acceptance of species protection. Therefore, any such measures would require transparent legal safeguards, strict administrative control, and clear conservation priorities.

Adaptive management also has social implications. Increasing wildlife populations may contribute to more frequent human–wildlife interactions, influencing public perception and the acceptance of conservation policies [9,17]. Therefore, conservation strategies should balance ecological, social, and economic considerations.

The analysis indicates that species such as European bison, moose, and beaver are currently subject not only to protection but also to varying forms of management within human-influenced ecosystems [4,13]. This requires continuous monitoring and adaptive decision-making. Overall, contemporary conservation does not rely solely on prohibitions but increasingly involves active and informed management. Effective protection, therefore, depends not only on legal frameworks but also on their practical implementation and alignment with ecological knowledge [10].

#### De Lege Lata and Amendment (De Lege Ferenda) Postulates

The research suggests that changes to the legal framework for the discussed species may be considered, although not all options are equally appropriate. Such changes may involve either modifying existing laws or adjusting how they are interpreted in practice, without changing their wording. In particular, the authors do not support any reduction of the legal protection of the European bison or its reclassification as a typical game species. At the same time, certain complementary solutions, such as the development of controlled farm breeding—similar to practices applied to American bison—may be considered within existing legal frameworks. In the case of the Eurasian beaver, hunting for the purpose of meat acquisition does not appear to be a primary management objective; however, the use of meat obtained as a result of legally authorized population control measures may be taken into account. As noted, such interventions are limited to exceptional cases and require administrative decisions by the competent environmental authorities.

The situation of the Eurasian moose differs in several respects. Formally, it is classified as a game species under Polish law; however, a long-standing moratorium has resulted in a practical suspension of hunting. Consequently, the current unavailability of moose meat on the market is primarily linked to regulatory and policy decisions rather than to statutory species protection. Any potential changes in this regard would therefore require careful consideration within the existing legal framework. Regardless of the management model applied, meat from moose intended for human consumption must originate from lawful sources and comply with all relevant veterinary and food safety requirements [20,26,27,28,29,32,33].

The legal situation regarding the acquisition of meat from protected species is complex and open to different interpretations. It may be based either on the literal wording of legal provisions or on their broader purpose.

Art. 52 of the Nature Conservation Act [18] prohibits the acquisition, possession, and preparation of specimens. In its literal meaning, this provision primarily concerns specimens used for display or utility purposes (e.g., taxidermy or trophies), rather than meat intended for human consumption. Extending this prohibition to meat would therefore require a broader, functional interpretation. At the same time, the Act also restricts the sale and transport of specimens, raising questions about the possibility of placing such products on the market.

From a purposive perspective, species protection primarily aims to safeguard populations rather than regulate all possible uses of animal products [53,85]. In this context, it may be argued that the use of meat obtained from legally justified actions does not necessarily conflict with conservation objectives, provided that it does not negatively affect the species.

According to the principles of legal interpretation, broader methods (such as teleological or functional interpretation) should be applied only when the literal wording is insufficient. In this case, the linguistic interpretation of Art. 52 may be considered adequate.

Consequently, it may be considered possible for competent authorities to authorize population control measures while also allowing the use of resulting by-products, including meat, on a case-by-case basis. Such use would remain strictly linked to legally justified actions and subject to existing regulatory controls.

In this context, it may be considered whether clarifying statutory provisions—particularly Art. 52 and 56 of the Nature Conservation Act [18]—could improve legal certainty. Any such changes would require careful consideration and should remain consistent with conservation objectives.

Similarly, the possibility of developing controlled European bison breeding may be explored within the existing legal framework, provided it does not adversely affect the species’ conservation status.

## 5. Conclusions

The current research demonstrates the close relationship between legal frameworks for species protection, wildlife management practices, and the ecological condition of protected animal populations. These factors include population size and structure, distribution, health status, and genetic diversity. Maintaining a balance between them requires adaptive and scientifically informed management approaches.

For all three species studied in this paper, the use of meat from animals legally removed for justified reasons could be considered within carefully regulated adaptive management strategies. This approach could be linked to zero-waste principles and sustainable resource management. However, it would also raise important legal, ethical, ecological, and social concerns and would require careful regulation and monitoring. The question of whether strict species protection can coexist with limited and strictly regulated consumption of meat from certain animal species requires further interdisciplinary research and careful legal, ecological, and ethical discussion.

## Figures and Tables

**Table 1:** Comparative overview of selected ecological characteristics, conservation benefits, management challenges, and socio-economic impacts associated with European bison, Eurasian beaver, and Eurasian moose populations in Poland.

Species	Estimated Population in Poland	Selected Ecological and Conservation Benefits	Main Management Challenges	Examples of Socio-Economic Impact
European bison (*Bison bonasus*)	ca. 3000 [1,3]	restoration of an endangered native species, maintenance of open habitats, and symbolic conservation value [11,12,13,14]	disease control, population fragmentation, human–wildlife conflicts [1,13,46]	crop damage, sanitary interventions [3,46]
Eurasian beaver (*Castor fiber*)	ca. 148,000 [3,7]	water retention, habitat creation, increased biodiversity, ecosystem engineering [5,16,47]	infrastructure conflicts, local overabundance [4,15,48]	flooding, embankment, and tree damage [3,6,49]
Eurasian moose (*Alces alces*)	>30,000 [3,9]	restoration of native megafauna, influence on vegetation structure and wetland ecosystems [9,17]	road safety, habitat expansion, population regulation [9,17,50]	traffic accidents, forestry and agricultural damage [9,10,51]

## Data Availability

The original contributions presented in the study are included in the article; further inquiries can be directed to the corresponding author.

## Abbreviations

The following abbreviations are used in this manuscript:

Art.	Article
CJEU	Court of Justice of the European Union
